# Ab Initio Molecular Dynamics Insight to Structural Phase Transition and Thermal Decomposition of InN

**DOI:** 10.3390/ijms25158281

**Published:** 2024-07-29

**Authors:** Jacek Piechota, Stanislaw Krukowski, Bohdan Sadovyi, Petro Sadovyi, Sylwester Porowski, Izabella Grzegory

**Affiliations:** Institute of High Pressure Physics, Polish Academy of Sciences, 29/37, Sokolowska Street, 01-142 Warsaw, Poland; stach@unipress.waw.pl (S.K.); bsad@unipress.waw.pl (B.S.); pedro@unipress.waw.pl (P.S.); sylvek@unipress.waw.pl (S.P.)

**Keywords:** indium nitride, ab initio, p-T phase diagram, thermal decomposition, N_2_ molecule

## Abstract

Extensive ab initio density functional theory molecular dynamics calculations were used to evaluate stability conditions for relevant phases of InN. In particular, the p-T conditions of the thermal decomposition of InN and pressure-induced wurtzite–rocksalt solid–solid phase transition were established. The comparison of the simulation results with the available experimental data allowed for a critical evaluation of the capabilities and limitations of the proposed simulation method. It is shown that ab initio molecular dynamics can be used as an efficient tool for simulations of phase transformations of InN, including solid–solid structural transition and thermal decomposition with formation of N_2_ molecules. It is of high interest, because InN is an important component of epitaxial quantum structures, but it has not been obtained as a bulk single crystal. This makes it difficult to determine its basic physical properties to develop new applications.

## 1. Introduction

Indium Nitride (InN) is an intriguing and very important, although relatively less known, semiconductor. Its importance is related to revolutionary development of InGaN-based light sources—Light Emitting Diodes and Laser Diodes [1], awarded the Nobel Prize in physics in 2014 [2]. Until 2002, InN had been considered as a wide bandgap (about 2 eV) member of the III-N semiconductor family, which includes GaN, AlN, and BN. Due to significant improvements in high purity molecular beam epitaxy (MBE) of InN, it was convincingly demonstrated that the bandgap of high structural quality pure InN is as low 0.64 eV [3,4,5]. Therefore, the energy spectrum covered by III-N semiconductors: *E*_g_(InN) = 0.64 eV, *E*_g_(GaN) = 3.47 eV [6,7], *E*_g_(AlN) = 6.08 eV [8] and *E*_g_(wBN) = 6.39 eV [9] became even more interesting, extending from the far infrared to ultraviolet spectral range. Moreover, the electron effective mass m*(InN) = 0.07*m*_0_ [4], corresponding to the “new” energy gap is much smaller than that of the other nitrides: GaN − m*(GaN) = 0.20*m*_0_ or AlN − m*(AlN) = 0.32*m*_0_ [4]. It indicates the possibility of hosting very high mobility electron gas in the InN crystal. However, the growth of InN single crystals is still a challenge due to specific thermodynamic properties of this nitride.

InN crystal is relatively soft, despite containing atomic nitrogen. The InN bonding is less covalent and more ionic, as well as much weaker than in the other nitrides reflected in larger lattice constants and the much smaller bandgap in InN [4]. Therefore, as you would expect, the thermodynamic properties of the In–N system are affected by both strong triple bonding in the N_2_ molecule, lowering the free energy of the In+N_2_ system, and the relatively weak bonding of the InN crystal [10,11]. That leads to very high pressures of nitrogen at relatively low temperatures along the InN(s)–In(l)–N_2_(g) triple phase stability line [12]. In addition, the solubility of nitrogen in liquid indium is extremely low, so the growth of InN bulk crystal from indium solution is difficult [12,13].

As a consequence, large bulk crystals of InN have not been grown so far and, due to the lack of high quality reference material, experimental investigations of exciting physical properties of InN are difficult.

An interesting route facilitating the efforts towards crystallization of InN and also providing valuable insight into the physical properties of the InN(s)–In(l)–N_2_(g) system could be obtained from ab initio molecular dynamics (MD) simulations. In this study, we use the ab initio MD simulations as an efficient tool to evaluate phase behavior of InN in wide pressure and temperature ranges. It can be useful for further exploration of phase diagram of InN: its experimental verification and its use as a background for crystal growth. An additional, encouraging motivation was a very reasonable agreement of the proposed approach with the experimental data we achieved recently for GaN [14].

### Phase Diagram of InN

**Figure 1:**
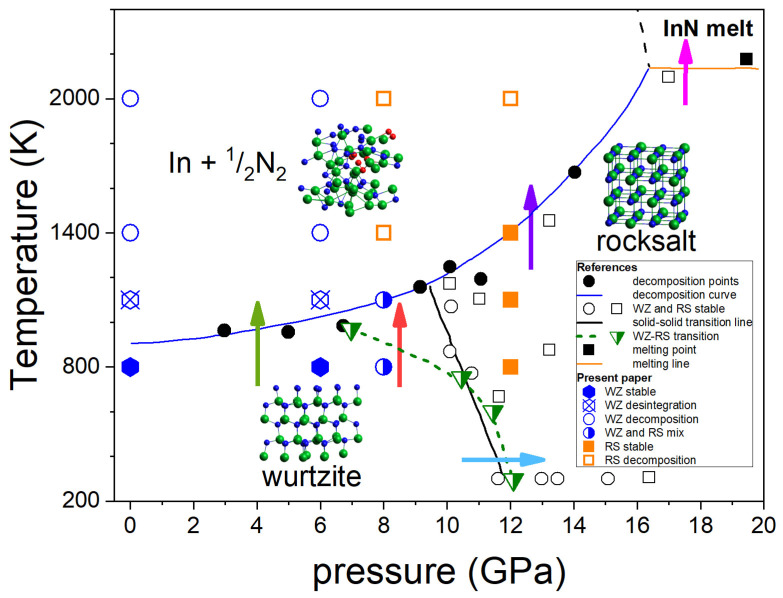
Phase diagram of InN. Experimental data from Saitoh et al. [15]: black solid circles and blue solid line—InN decomposition points and line; black open circles and squares—stable InN WZ and InN RS respectively; black solid square—InN melting; solid orange line—InN melting line. Experimental data from Soignard et al. [16]: green semi-solid inverted triangles—InN WZ-RS transition points, green dashed line—guide for eyes. Arrows: phase transitions as described in the text. The theoretical results of this work are indicated by symbols as described in the inserted legend.

Figure 1 summarizes the most consistent experimental data on the InN p-T phase diagram obtained by Saitoh et al. [15] and Soignard et al. [16] based on the in situ X-ray diffraction (XRD) measurements at high pressure–high temperature conditions.

The diagram shown in Figure 1 indicates the following behavior of InN:

1. At the lower pressure range (up to 7–10 GPa), InN decomposes when heated above approx. 710 °C [9] (green arrow in Figure 1). It was also confirmed by our earlier differential thermal analysis (DTA) experiments up to 2 GPa [13] at high pressure of N_2_ gas. The independence of decomposition temperature on pressure suggests that the equilibrium temperatures in the InN–In–N_2_ system can be even lower than the measured ones.
2. At 12 GPa (low T) to 7–10 GPa (high T), a structural phase transition from hexagonal wurtzite to cubic rocksalt phase induced by increasing pressure is observed (blue arrow in Figure 1). The borderline between the two solid phases is inclined towards lower pressures; however, the different linear or non-linear character of this line follows from XRD experiments reported in [15,16], respectively. A possible verification could be checking if at, i.e., 8 GPa the InN crystal in its wurtzite phase transforms into rocksalt at heating to 800–1000 K (red arrow in Figure 1).
3. The InN crystal in its high pressure rocksalt phase also decomposes at high temperatures, but the decomposition temperature strongly increases with increasing pressure (violet arrow in Figure 1).
4. The decomposition is suppressed only at pressure as high as >16 GPa and then the congruent melting of InN (without formation of N_2_, thus reversible) is possible (magenta arrow in Figure 1).

In the next sections, we explore by the ab initio MD simulations the following phase transitions of InN:

- wurtzite-to-rocksalt structural phase transition induced by high pressure (blue arrow);
- wurtzite-to-rocksalt structural phase transition induced by high temperature at 8 GPa (red arrow);
- thermal decomposition of InN crystal in both wurtzite and rocksalt phases (green and violet arrow, respectively).

In this paper, special attention is given to the determination of the p-T stability range of InN in its wurtzite modification, which is particularly important for the design of future crystallization experiments in high-pressure reactors with high volumes, routinely used for diamond or BN growth. Congruent melting of InN (magenta arrow in Figure 1) was not considered.

## 2. Result and Discussion

### 2.1. Pressure Induced Solid–Solid Phase Transition at Low Temperature

**Figure 2:**
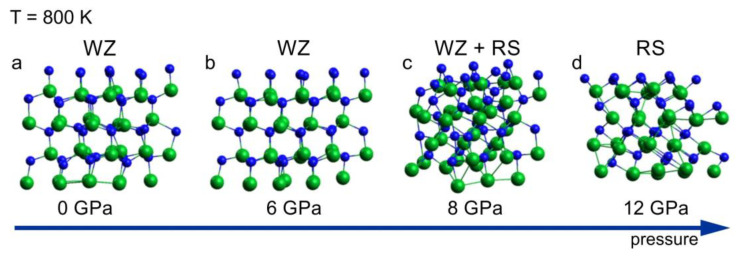
InN wurtzite cluster after 6 ps virtual annealing at 800 K at different pressure: (**a**) 0 GPa, (**b**) 6 GPa, (**c**) 8 GPa and (**d**) 12 GPa; green balls—indium atoms, blue balls—nitrogen atoms. The arrow represents the increase of the pressure.

A sequence of the InN clusters annealed at 800 K for a period of 6 ps, at different pressure, is shown in Figure 2. The sequence illustrates the wurtzite–rocksalt structural phase transition, starting at pressure of 8 GPa. The structure identification is based on the analysis of coordination numbers in the cluster presented in Figure 2c, in which both 4 fold (wurtzite) and 6 fold (rocksalt) coordination spheres were found. The transition pressure of 8 GPa is lower than the one following from the high pressure XRD experiments (10–11 GPa) [15,16]. The possible causes for this disagreement are hidden in both theoretical and experimental approaches. The mixed coordination observed at the transition point (8 GPa, 800 K) suggests a “nucleation–growth” mechanism of the structural transformation. Therefore, a hysteresis is expected, which is indeed observed in the experiments, making the relation between theoretical and experimental transition pressure values consistent. Moreover, intrinsic errors in both the simulations and the measurements have to be taken into account.

According to the simulation, at 12 GPa, the crystal is fully converted into its high-pressure rocksalt phase (Figure 2d), as confirmed by the coordination analysis, which is in agreement with the experimental data. The root mean square deviation (RMSD) of average atomic positions as a function of time *t* indicates whether the system departs from the reference structure during MD simulation. The following statistics were obtained:

1. A RMSD ≤ 1.0 Å if no phase transition takes place;
2. A 2.0 Å *<* RMSD *<* 3.0 Å in the case of structural phase transition (e.g., wurtzite → rocksalt);
3. A RMSD ≥ 3.0 Å in the case of melting of the system.

Since the NVT Canonical Ensemble has been used in the simulation, where pressure was a function of the NVT set, and remembering that the pressure induced wurtzite– rocksalt phase transition includes a significant change in the InN volume [15], the pressure trajectory during the simulation has to be analyzed. The analysis for the wurtzite–rocksalt transition during 6 ps indicated an averaged pressure decrease not bigger than 300 MPa at intrinsic pressure value scattering of ±500 MPa.

### 2.2. Temperature Induced Solid–Solid Phase Transition at 8 GPa

**Figure 3:**
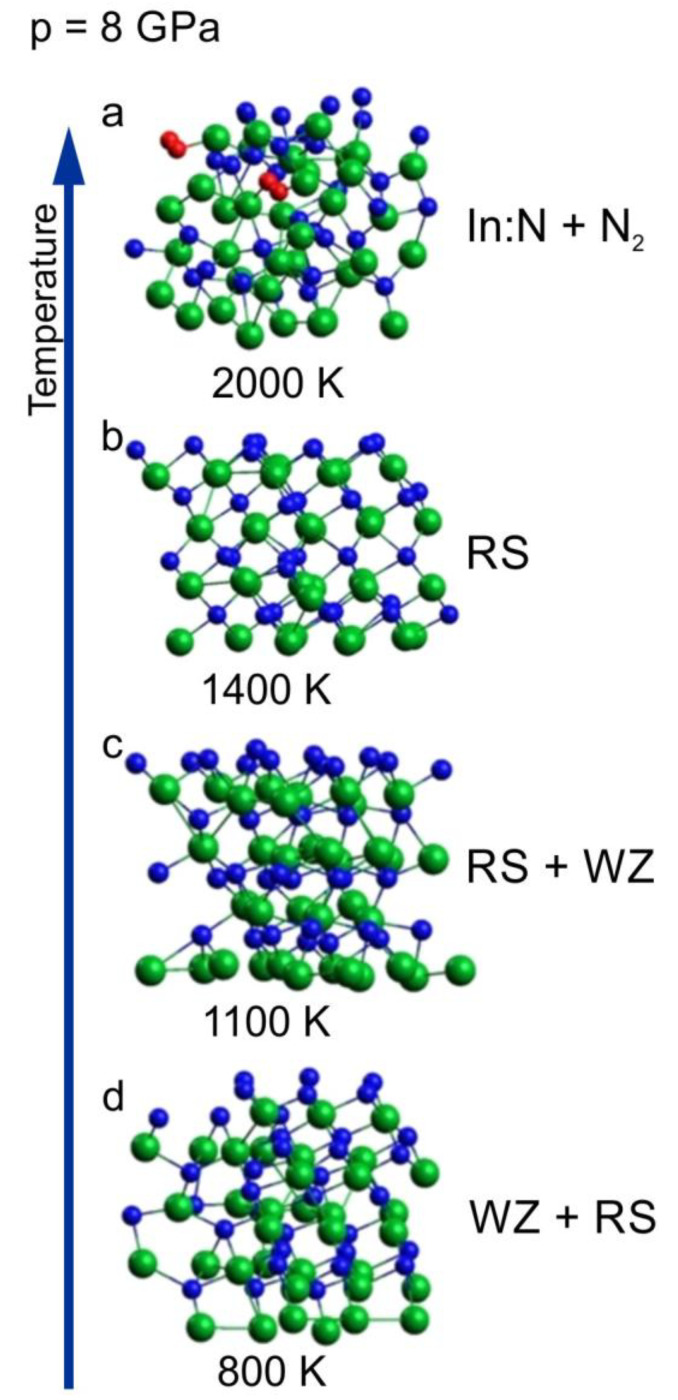
InN wurtzite cluster after 6 ps time evolution virtual annealing at 8 GPa and at different temperatures: (**a**) 800 K, (**b**) 1100 K, (**c**) 1400 K, (**d**) 2000 K; green balls—indium atoms, blue balls—nitrogen atoms, red balls—nitrogen atoms forming N_2_ molecules. The arrow represents the increase of the temperature.

**Figure 4:**
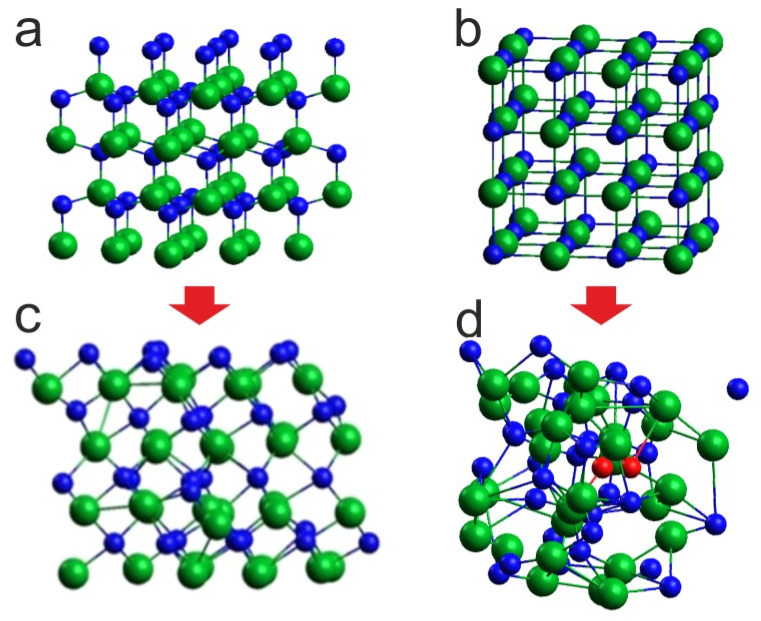
Effect of 6 ps virtual annealing at 8 GPa and 1400 K on the InN cluster: (**c**) with initial wurtzite supercell (**a**), (**d**) with initial rocksalt supercell (**b**); green balls—indium atoms, blue balls—nitrogen atoms, red balls—nitrogen atoms forming N_2_ molecules. The arrows represent the transformation during simulation.

In Figure 3, we have shown a sequence of clusters corresponding to 8 GPa and different temperatures ranging from 800 to 2000 K. The initial supercell was wurtzite (Figure 4a).

The sequence starts from the cluster with mixed coordination, which was already presented in Figure 3c as a border case between wurtzite and rocksalt phases at 800 K. With increasing temperature (1100 K and 1400 K), an ordering towards cubic rocksalt phase is clearly visible, which confirms that the borderline between the two solid phases is negatively inclined and rather non-linear, as was suggested in [16].

For the highest temperature of 2000 K, the crystalline order is lost and the crystal decomposes with the formation of N_2_ molecules (decomposition process is analyzed in the next sub-section).

Given that at 8 GPa and 1100 K the phase of InN clearly evolves towards rocksalt, we decided it would be more consistent for 1400 K and 2000 K, to start the simulations from the initial supercell in the rocksalt structure (Figure 4b). The corresponding comparison for 1400 K is shown in Figure 4.

The situations are quite different: for the initial supercell in wurtzite, its conversion to the rocksalt structure with no signs of lattice disintegration is visible, whereas for the initial supercell in rocksalt, the lattice is thermally destabilized and, moreover, N_2_ molecules start to emerge, which is in agreement with the experimental observations. This indicates that, for the applied simulation algorithm and time scale, it is reasonable to start the virtual heating of the crystal, avoiding the structural phase transition during the thermal evolution of the system.

### 2.3. Decomposition of InN

The experimental diagram of InN phase stability (metastability?) shown in Figure 1 indicates that the crystal should decompose at high temperatures to indium and N_2_ at pressure up to approx. 16 GPa. In the XRD experiments [15], the thermal decomposition was reflected by irreversible disappearance of the XRD spectral features of the InN crystalline phases. In contrast, at a pressure of 19.2 GPa, the XRD spectrum corresponding to the rocksalt InN crystal lattice also disappeared; however, at cooling, its recovery has been observed. Therefore, it was suggested that there was a sign of congruent melting of InN, and that the congruent melting of InN is possible only in for the high pressure rocksalt phase, due to the wurtzite InN decomposition before reaching its melting point in the whole pressure range of the wurtzite InN stability.

**Figure 5:**
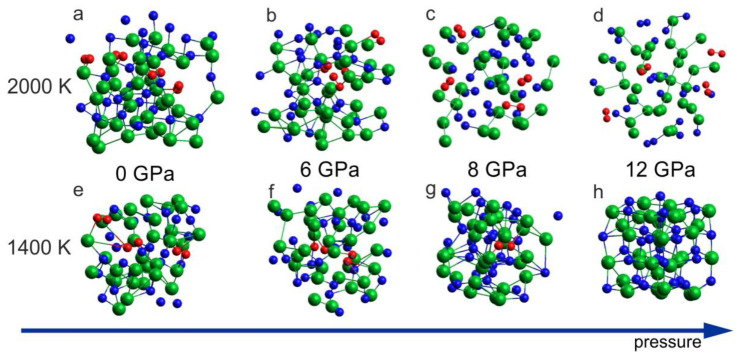
Effect of 6 ps virtual annealing at 2000 K (**a**–**d**) and 1400 K (**e**–**h**) on the InN cluster at different pressures: (**a**,**e**) 0 GPa (initial supercell: wurtzite); (**b**,**f**) 6 GPa (initial supercell: wurtzite); (**c**,**g**) 8 GPa (initial supercell: rocksalt); (**d**,**h**) 12 GPa (initial supercell: rocksalt). The arrow represents the increase of the pressure.

The ab initio MD simulations reproduced the thermal decomposition of InN in terms of the crystal lattice destruction and formation of N_2_ molecules. Figure 5a–d includes the results for 2000 K, which demonstrates formation of the N_2_ dimers for all considered pressure conditions: 0 GPa, 6 GPa, 8 GPa, and 12 GPa. At a lower temperature, T = 1400 K, the decomposition with N_2_ formation was observed for 0 GPa, 6 GPa and 8 GPa, but for 12 GPa, the formation of N_2_ molecules was suppressed (Figure 5h) and the rocksalt structure was preserved. This suggests that, at T = 1400 K, the InN crystal (rocksalt) is stable at p = 12 GPa and unstable at p = 8 GPa, which is in a very good agreement with experimental suggestions illustrated in Figure 1.

**Figure 6:**
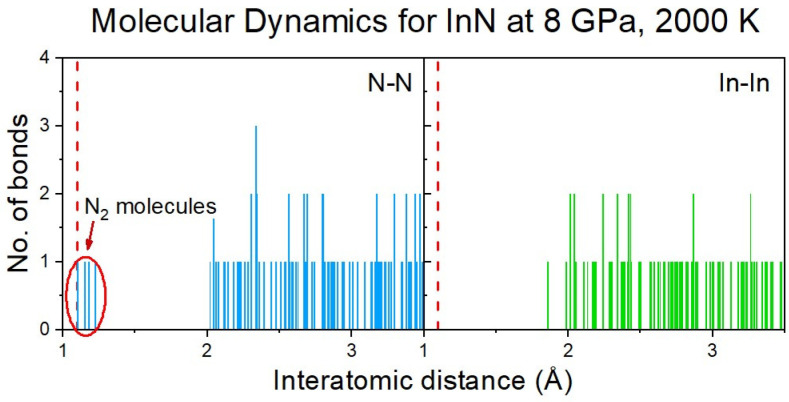
Distribution of N-N and In-In (for comparison) interatomic distances in the InN cluster at 8 GPa and 2000 K (initial supercell: rocksalt). A tendency to formation of N_2_ molecules is clearly visible.

The formation of N_2_ molecules was identified via the analysis of the distribution of the interatomic distances between the nitrogen atoms (Figure 6), and further confirmed by the Crystal Orbital Hamilton Population (COHP) analysis of the selected pairs of nitrogen atoms [17,18].

## 3. The Simulation Method

Temporal evolution by the ab initio MD simulations requires a huge amount of calculations, which is challenging because, at each time step, the solutions of full quantum mechanical problems via density functional theory (DFT) Kohn–Sham equations have to be obtained [19]. The forces acting on each atom in the system, are obtained via Hellmann– Feynman theorem used for time integration of MD time evolution equations [20]. The standard time step, 0.2 fs long, was employed in this study. Total simulation time lasted 30,000 steps, and was carried out for each p,T set. The electron temperature in the Fermi–Dirac distribution was increased to accelerate the transformation of the structure [21,22]. This does not affect the ionic temperature, as electron–phonon interaction is not included in the model [22,23]. For the time-dependent MD calculations, the optimal choice is Verlet integration algorithm [20], which is precise to the fourth order in the time step length. This algorithmically simple method requires a single evaluation of the force acting on each atom at every time step; thus, it is the most effective numerically. The Verlet algorithm is implemented in the time-dependent simulations in the Spanish Initiative for Electronic Simulations of Thousands of Atoms (SIESTA) DFT package selected for our simulations [24]. The solution of nonlinear Kohn–Sham equations is expressed as sum of a finite radius pseudo-atomic orbital functions set [25] that is reduced in number by application of the Troullier–Martins norm-conserving nitrogen and indium pseudopotentials in the Kleinman–Bylander formulation [26,27]. The exchange-correlation energy was approximated by the Perdew–Burke–Ernzerhof functional developed for solid and surface calculations (PBEsol) [28]. The solutions of the Kohn–Sham nonlinear equations were obtained by self-consistent field (SCF) loop, terminated when the maximum difference between two consecutive values of all elements of the density matrix fell below 10^−4^.

In the reported simulations, the finite size InN 3 × 3 × 2 supercell was used containing total 72 indium and nitrogen atoms, arranged in the hexagonal wurtzite lattice. In some cases, the initial arrangement of In–N atoms in the ideal cubic rocksalt lattice stable at high pressures [15,16], was used intentionally. Periodic boundary conditions were imposed for atom motion, pseudopotentials, and electric potential. For the considered systems, the Brillouin zone k-integration was replaced by sum over 3 × 3 × 3 Monkhorst–Pack k-point mesh. The initial supercells used in our study are presented in Figure 4a,b.

The parameterization of the equations and solution basis was verified by direct comparison to the experimental data for wurtzite InN, gaseous N_2_, and pure In. The ab initio lattice parameters of wurtzite InN are $a_{\mathrm{InN}}^{\mathrm{DFT}}$ = 3.533 Å and $c_{\mathrm{InN}}^{\mathrm{DFT}}$ = 5.693 Å, which remain in a very good agreement with the X-ray data: $a_{\mathrm{InN}}^{\exp.}$ = 3.5374(1) Å and $c_{\mathrm{InN}}^{\exp.}$ = 5.7027(5) Å [29], respectively.

The calculated dissociation energy and bond length of the N_2_ molecule were $\Delta E_{\mathrm{diss}.}^{\mathrm{DFT}}$(N_2_) = 9.801 eV and $d_{N-N}^{\mathrm{DFT}}$ = 1.092 Å, in good agreement with the experimental $\Delta E_{\mathrm{diss}.}^{\exp.}$(N_2_) = 9.790 eV [30] and $d_{N-N}^{\exp.}$ = 1.097 Å [31], respectively.

The properties of metallic indium were evaluated including density and cohesive energy. The ab initio energy of In atomization was $\Delta E_{\mathrm{DFT}}^{\mathrm{atom}}$(In) = 2.39 eV/atom, close to the experimental value of vaporization enthalpy $\Delta E_{\exp.}$ = 2.35 eV/atom [32]. The density of the In liquid at the melting temperature T_m_(In) = 156.6 °C, is *ρ*_exp._(In) = 7.02 g/cm^3^, which is comparable with the ab initio simulation result: *ρ*_DFT_(In) = 7.05 g/cm^3^.

For the time-integration procedure in the imposed NVT conditions, i.e., corresponding to the Canonical Ensemble, the Nosé–Hoover thermostat for temperature stabilization [33,34] was employed. From the initial setup of atoms in the form of the wurtzite/rocksalt InN lattice (Figure 1), the target temperature was achieved by rescaling the velocities of all atoms to the kinetic energy obtained from equipartition principle for a selected temperature T. The molecular pressure was determined using the Irving–Kirkwood formula [35]. More details related to the ab initio MD simulations of nitrides may be found in Ref. [14]. The pressure–temperature conditions used in this study are included into Figure 1.

## 4. Conclusions and Outlook

The reported ab initio MD approach simulates both the pressure- and temperature induced structural phase transitions of InN. The results suggest that the wurtzite–rocksalt borderline is negatively inclined and nonlinear, as follows from [16] but not supported by [15]. The ultimate experimental verification of this controversy is strongly needed for a precise evaluation of the wurtzite InN stability range. Then, the optimum temperature and corresponding pressure can be found and explored for crystallization of the nitride.

Interestingly, the thermal decomposition of InN with the formation of N_2_ molecules was identified in the simulation, which is a great advantage of the ab initio MD. The approach could be especially useful for the evaluation of the still-unknown melting curve of InN, for which suppression of the N_2_ formation is expected. For high temperatures (≥1400 K) expected for melting, the relatively short simulation time of 6 ps should be sufficient to complete the relevant transitions. For the InN melting experiments, the laser heated diamond anvil cell technique, coupled with XRD, EXAFS, or Raman spectroscopy, as in [36], will be used.

## Data Availability

The raw data supporting the conclusions of this article will be made available by the authors upon reasonable request.
